# Occupation and working outcomes during the Coronavirus Pandemic

**DOI:** 10.1007/s10433-021-00651-5

**Published:** 2021-10-08

**Authors:** Agar Brugiavini, Raluca E. Buia, Irene Simonetti

**Affiliations:** Department of Economics, University Ca’ Foscari of Venice, Venice, Italy

**Keywords:** Pandemic, Work interruption, Essential/unessential jobs, Remote work, Social interaction, J01, J21, J24

## Abstract

Using data from the first wave of the SHARE COVID-19 Survey and additional information collected from the previous waves of SHARE (Survey of Health Ageing and Retirement in Europe), we explore the effects of job characteristics on two outcomes: (i) the probability of work interruptions and (ii) the length of such interruptions during the first phase of the Coronavirus Pandemic. In order to assess the relationship between job features and labour market outcomes, we define two indexes proxying the pre-COVID-19 technical *remote work feasibility* as well as the level of *social interaction* with other people while working. Moreover, we use an indicator that classifies ISCO-08 3-digit job titles based on the *essential* nature of the good or service provided. We find that job characteristics have been major determinants of the probability of undergoing work interruptions and their duration. In addition, we show that women have been negatively affected by the Pandemic to a much larger extent than men, suggesting the relevance of the intrinsic characteristics of jobs they are mainly involved in, and the role of gender selection into specific activities. Not only females were more likely to have undergone work interruptions but they also exhibited larger probabilities of longer work breaks. A similar impact is seen for self-employed and less-educated workers.

**Supplementary Information:**

The online version contains supplementary material available at 10.1007/s10433-021-00651-5.

## Introduction

The outbreak of the COVID-19 Pandemic at the beginning of 2020 led to radical changes in many aspects of individuals’ lives. Mitigation policies, based on limiting social contacts and physical distancing, implied suspension, reduction and/or converting several activities, including work, to remote mode. As shown by a series of indicators (OECD, Eurostat, 2020), the lockdown measures had enormous negative economic effects as well as changing several aspects of life, from the labour market activities to individuals’ health and social behaviour. The available macroeconomic evidence documents a dramatic increase in unemployment (OECD, 2020a, 2020b) in spite of the joint efforts of governments and firms to prevent work interruptions by fostering — when possible—home working/teleworking (especially at the very beginning of the Pandemic) or by rearranging working spaces to maximize the physical distance. The OECD and ILO publications on employment trends indicate that low-qualified workers, individuals engaged in the informal economy, immigrants and women are the most vulnerable groups.

In the effort to identify the job-related drivers behind the negative effects of social distancing measures and mobility restrictions, the recent literature has focused on jobs that can be performed from home (WFH). Dingel and Neiman (2020) analyse occupations traits in the USA starting from the O*NET dictionary of occupations, while Yasonev (2020) investigates workers’ characteristics, showing that young, low-educated and low-wage workers, as well as ethnic minorities and immigrants, are less likely to have jobs suitable for home working. Cetrulo et al. (2020) make use of the Italian INAPP-ICP data and find that marked occupational inequalities may result from the lockdown restrictions, with a high concentration of WHF jobs among managerial and executive categories, academics, technical professionals and clerical support workers as opposed to sales and service workers, manual operators, artisans and elementary occupations. In a cross-country study, Boeri et al. (2020) report that the percentage of jobs that can be performed remotely differs among European countries, from 23.95% in Italy to 31.38% in UK. A related line of investigation, developed before the Pandemic outbreak, has focused on some essential features of the tasks performed—(i) abstract, (ii) routine and (iii) manual—in order to explain occupational differences among workers (Autor and Dorn 2009; Autor and Dorn 2013; Deming 2017).

This evidence suggests the existence of high heterogeneity in measuring the consequences of the Coronavirus Pandemic on the labour market, which is partly due to general labour market conditions in a given country, partly to socioeconomic conditions and largely due to intrinsic characteristics of the job performed. Therefore, individual level data —especially on each job—are a crucial requirement to disentangle the role of these determinants. This paper investigates to what extent the type of occupation —and its peculiarities — drove individual's labour market consequences during the first wave of the Pandemic. Our analysis is relevant as it allows identifying workers who experienced the worst economic penalties due to the sanitary emergency, the most “vulnerable” activities. The interruption of working activity —either temporary or permanent — led to sizeable losses in terms of income, especially during the first phase of the Pandemic. Moreover, the longer the duration of the work interruption, the more likely it is to turn into "permanent" unemployment status at the end of the crisis, especially for the older age groups.

Recent data collected by the SHARE COVID-19 Survey allows for a detailed study of the changes in working conditions experienced by individuals aged 50 and over, as it contains information about respondents both before and during the COVID-19 outbreak. More precisely, we create a detailed dataset based on the pre-COVID-19 information available in panel format at the individual level in the ongoing SHARE survey, plus the information collected through the first wave of SHARE COVID-19 survey, and a classification of the occupations based on ISCO-08 3-digit codes.

Our approach is innovative as it deals with jobs traits allowing us to capture some crucial characteristics in a more parsimonious way. Based on Fasani and Mazza (2020), we first classify each ISCO-08 3-digit occupation according to the essential nature of goods and services provided. Moreover, by following Basso, Boeri, Caiumi, Paccagnella (2020) we generate two indexes for each code: (i) the remote work feasibility index measuring to what extent an occupation can be performed from home and (ii) the social interaction index assessing the intensity of social/physical contacts required in the workplace. These indexes are based on questions drawn from the Bureau of Labour Statistics (BLS) O*NET Survey data 2018, thus reflecting jobs features as if they were carried out in “normal times” (pre-COVID-19). In this way, we are able to distinguish between jobs that continued to be performed safely enough during the Pandemic because they usually involve a low level of social contact, and jobs that could potentially become safe because they suited remote working.

We model work continuity through an analysis in two steps. First, we estimate the effect of job characteristics on the probability of having experienced a temporary or permanent work interruption. Next, we assess the correlation between occupation traits and the length of interruptions. Our findings reveal that job characteristics have been major determinants of the probability of undergoing work interruptions as well as of their duration: unessential occupations are associated with a high prevalence of work interruptions, especially when unsuitable to be performed remotely or involve significant levels of social contacts. In addition, we show that women have been negatively affected by the Pandemic to a much larger extent than men. Females were more likely to experience work interruptions and longer work breaks. Our results point to the intrinsic characteristics of jobs performed by women, and the role of gender selection into specific activities, especially for female workers in older cohorts. Finally, we find that self-employed and less-educated workers display larger probabilities of work breaks and longer interruptions.

The paper is organized as follows: “Data” section presents the data and relevant questions of the SHARE COVID-19 questionnaire used in the analysis. “Empirical strategy” section describes the empirical specifications while “Results” section presents the results. “Conclusions” section concludes.

## Data

We use information from the first wave of the SHARE COVID-19 Survey to assess how working conditions of Europeans aged 50 and over evolved during the first wave of the Coronavirus Pandemic. The data collection was carried out three to six month after the Pandemic outbreak, therefore, it overlaps with lockdown periods in some countries and possibly with periods when the lockdown measures were already lifted in some others. Our analysis focuses on individuals who report to have been working (as employed or self-employed) at the time of the COVID-19 outbreak.^1^ Our final sample includes 7,619 people of which 44.30% are men, and 55.70% are women.^2^ Figure 1 in the supplementary material describes the sample composition by country and age groups.

**Fig. 1:**
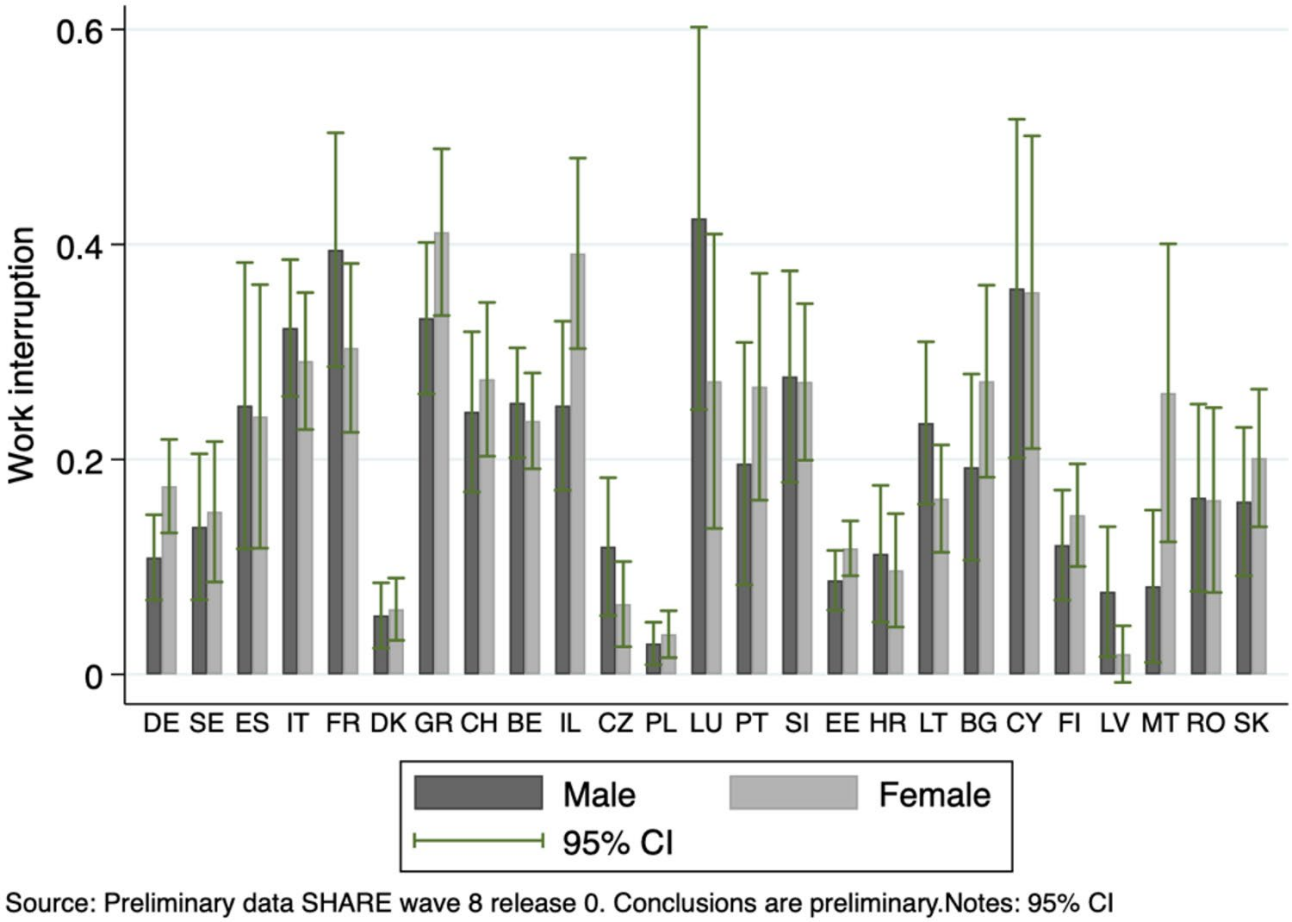
Fractions of work interruption by country and gender

### Working status during the Coronavirus Pandemic

A first outcome of interest to develop our research question is the event “work interruption” experienced by the respondents during the first wave of the Pandemic. This outcome is elicited through the question: “*Due to the Corona crisis have you become unemployed, were laid off or had to close your business?*”. Note that in this question respondents are instructed to answer “yes” also when they have only temporarily suspended their working activity. In order to estimate the parameters of interest, we define a categorical variable, which takes value one if the respondent reports work interruptions and value zero otherwise. The descriptive table provided in the supplementary material show that 18% of individuals in our sample declared a work interruption. Figure 1 shows the fraction of work interruptions by gender and country: significant heterogeneity emerges among countries and unconditional frequencies do not show any clear gender patterns.

The fraction of women who temporarily or permanently stopped their activity is particularly high in Israel and Greece but lower than for men in Luxembourg, Latvia and Lithuania. As we argued, in order to explain these patterns, one needs detailed information on the characteristics of the labour market and individual characteristics, including demographics and type of activities performed at work.

**Fig. 2:**
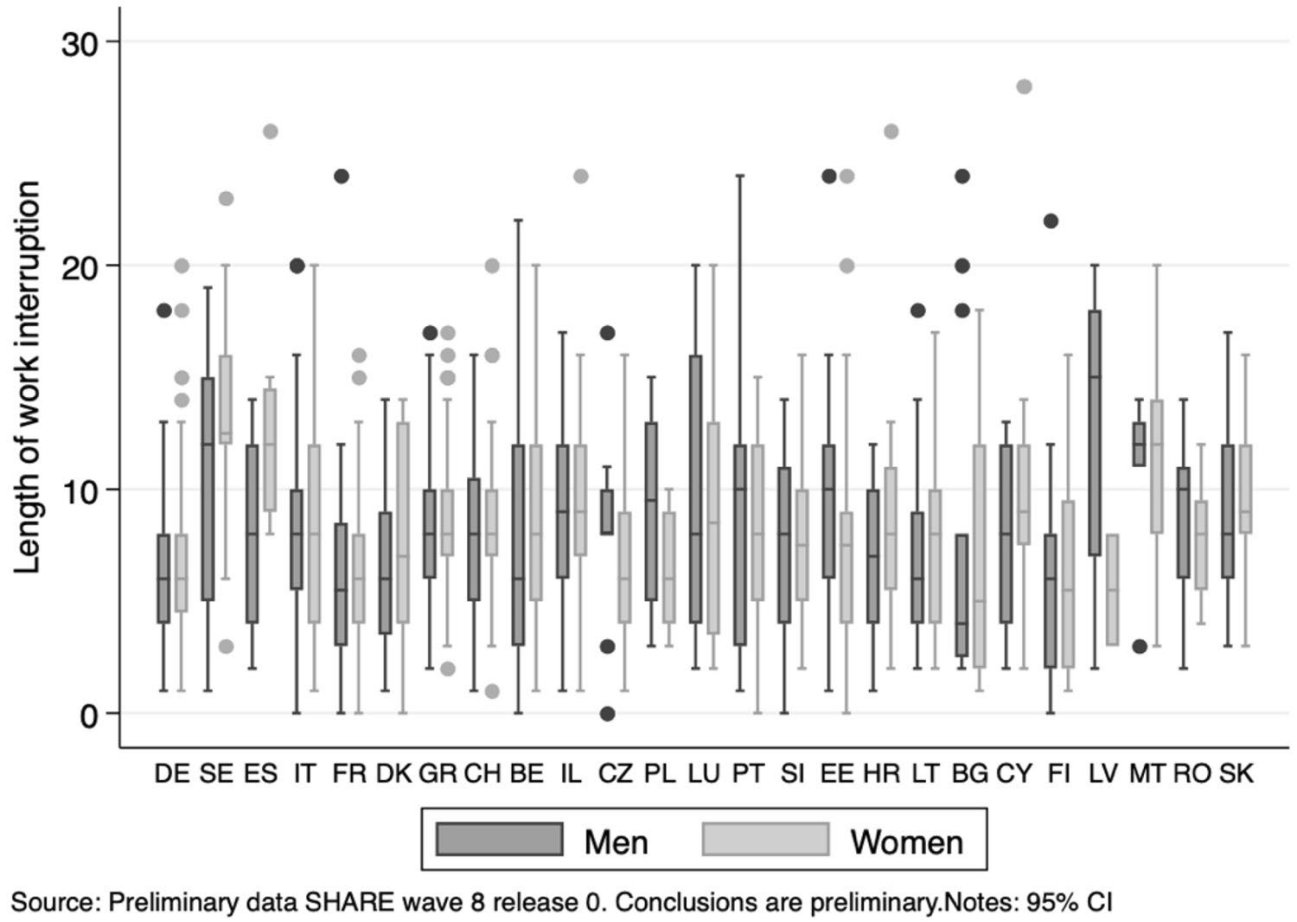
Length of work interruption by country and gender

A second outcome is the intensive margin: i.e. the length of a work interruption, based on the question: "*How long were you unemployed, laid off or had to close your business?*" –—measuring the number of weeks of interruption. This variable lends itself to different possible specifications, as we shall later explain. As a first approximation, we define a categorical variable taking three possible values: value zero if respondents continued working (82% of individuals), value one if they experienced a "short" interruption (around 11% of them experienced an interruption between 1 and 8 weeks) or value two if they stopped working for more than 8 weeks (7%).^3^ Figure 2 describes the distributions of the length of respondents’ work interruptions conditional on having undergone such events, by country and gender. The graph documents important differences between men and women—particularly strong in several countries such as the Czech Republic, Latvia, Spain or Sweden —as well as large heterogeneities among countries.

### The role of the job characteristics

The descriptive evidence provided in Fig. 1 outlines the major role of the Pandemic in changing individuals’ working patterns. Differences in labour market experiences could be related to multiple factors: partly to the stringency of lockdown measures in a given country, partly to pre-pandemic socioeconomic conditions and largely to the intrinsic characteristics of the job. In this paper, we build a unique dataset by combining the SHARE COVID-19 Survey with information from the regular SHARE survey up to wave 8.^4^ This process allows us to create a detailed dataset of work characteristics before and during the Pandemic. The novelty relates to the use of the ISCO-08 3-digit codes associated with the job performed that are collected for working respondents in waves 6 through to 8.^5^ This linkage provides us with a very large set of occupations from which we infer jobs characteristics, as well as details of tasks that workers are involved in.

However, potential drawbacks may arise from this wealth of information. Due to the density of the ISCO-08 codes (3-digit), the set of possible occupational titles is so wide that some categories may not be well-represented. Moreover, the use of a full set of indicators in pooled estimations may also translate into a loss in degrees of freedom due to the large number of explanatory variables that need to be included (“curse of dimensionality”). It is worth recalling that we are looking at a sample of Europeans aged 50 and over, meaning that job characteristics potentially relevant for younger workers may not apply in our study.

To overcome these issues—while taking advantage of the richness of such a detailed job classification—we exploit multiple aspects related to occupations characteristics. First of all, we classify jobs according to a dimension that was deemed relevant during the COVID-19 Pandemic: the *essential nature* of goods or services produced and provided. This variable identifies workers who perform crucial tasks, spanning from highly skilled professionals such as doctors to low-skilled workers, like food processers. More precisely, we take advantage of the list of ISCO-08 3-digit codes as identified by Fasani and Mazza (2020) and available in Table 1.^6^ Secondly, by following Basso et al. (2020) methodology, we built two indexes meant to measure two key features of occupations: (i) the extent to which an occupation could be technically performed from home and (ii) a measure of the level of social interaction when performing the job. Both indices are generated at an ISCO-08 3-digit level, using pre COVID-19 data elicited from the Bureau of Labour Statistics (BLS) O*NET Survey data 2018. Thus, it is worth emphasising that they are built on pre-pandemic characteristics, that is, the way jobs were carried out in “normal times”.^7^

**Table 1:** Essential jobs by ISCO-08 3-digit codes

ISCO-08 3-digit	Occupation
213	Life Science Professionals
214	Engineering Professionals (excluding Electrotechnology)
221	Medical Doctors
222	Nursing and Midwifery Professionals
223	Traditional and Complementary Medicine Professionals
224	Paramedical Practitioners
226	Other Health Professionals
231	University and Higher Education Teachers
232	Vocational Education Teachers
233	Secondary Education Teachers
234	Primary School and Early Childhood Teachers
235	Other Teaching Professionals
251	Software and Applications Developers and Analysts
252	Database and Network Professionals
311	Physical and Engineering Science Technicians
312	Mining, Manufacturing and Construction Supervisors
313	Process Control Technicians
314	Life Science Technicians and Related Associate Professionals
315	Ship and Aircraft Controllers and Technicians
321	Medical and Pharmaceutical Technicians
322	Nursing and Midwifery Associate Professionals
351	Information and Communications Technology Operations and User Support Technicians
352	Telecommunications and Broadcasting Technicians
511	Travel Attendants, Conductors and Guides
516	Other Personal Services Workers
531	Child Care Workers and Teachers’ Aides
532	Personal Care Workers in Health Services
611	Market Gardeners and Crop Growers
612	Animal Producers
613	Mixed Crop and Animal Producers
751	Food Processing and Related Trades Workers
816	Food and Related Products Machine Operators
831	Locomotive Engine Drivers and Related Workers
832	Car, Van and Motorcycle Drivers
833	Heavy Truck and Bus Drivers
835	Ships’ Deck Crews and Related Workers
911	Domestic, Hotel and Office Cleaners and Helpers
912	Vehicle, Window, Laundry and Other Hand Cleaning Workers
933	Transport and Storage Labourers
961	Refuse Workers

*Source*: Fasani and Mazza (2020)

The Bureau of Labour Statistics (BLS) O*NET Survey data 2018 provides a detailed description of work traits for each job in the USA (“work activities” and “work context”).^8^ Based on a broad range of questions, Basso et al. (2020) define four groups of occupations, capturing different “degrees of safeness” in the workplace and classify each ISCO-08 3-digit code accordingly. The most restrictive definition of safeness includes jobs potentially performed remotely (category 1). The second and third categories relax the previous definition and incorporate, besides the occupations in the first class, also jobs with a ‘low physical proximity and limited exposure to customers and to the public’ (category 2) and jobs with a “higher degree of interactions with external customers, but the level of physical proximity remains low” (category 3).^9^ The fourth group is a residual class—labelled as “unsafe jobs”—including all the remaining jobs with a relatively high risk of contagion. Although we use the same set of O*NET questions and thresholds, we do not stick to their definition of safeness. We completely separate the concepts of remote work feasibility and level of social interactions by defining two distinct indexes ranging between 0 and 1:

- The *remote work feasibility index* measures the extent to which a specific activity is suitable for remote work. We construct it using the same procedure and the same questions elicited by Basso et al. (2020) to describe the importance of physical/computer-based tasks in each job.^10^ A value equal to 0 means that a job cannot be performed at home (e.g. “primary school and early childhood teachers” (234), “medical doctors” (221) or “domestic, hotel and office cleaners and helpers” (911)), while a value of 1 is associated to ISCO-08 codes suited perfectly to home working from a technical point of view (e.g. “finance professionals” (241) or “legal professionals” (261)).
- The *social interaction at work* index proxies the level of physical and social interaction with other people while working. It is built on questions regarding the physical proximity to other persons, the importance of interactions with the public and the frequency of exposure to diseases or infection.

**Fig. 3:**
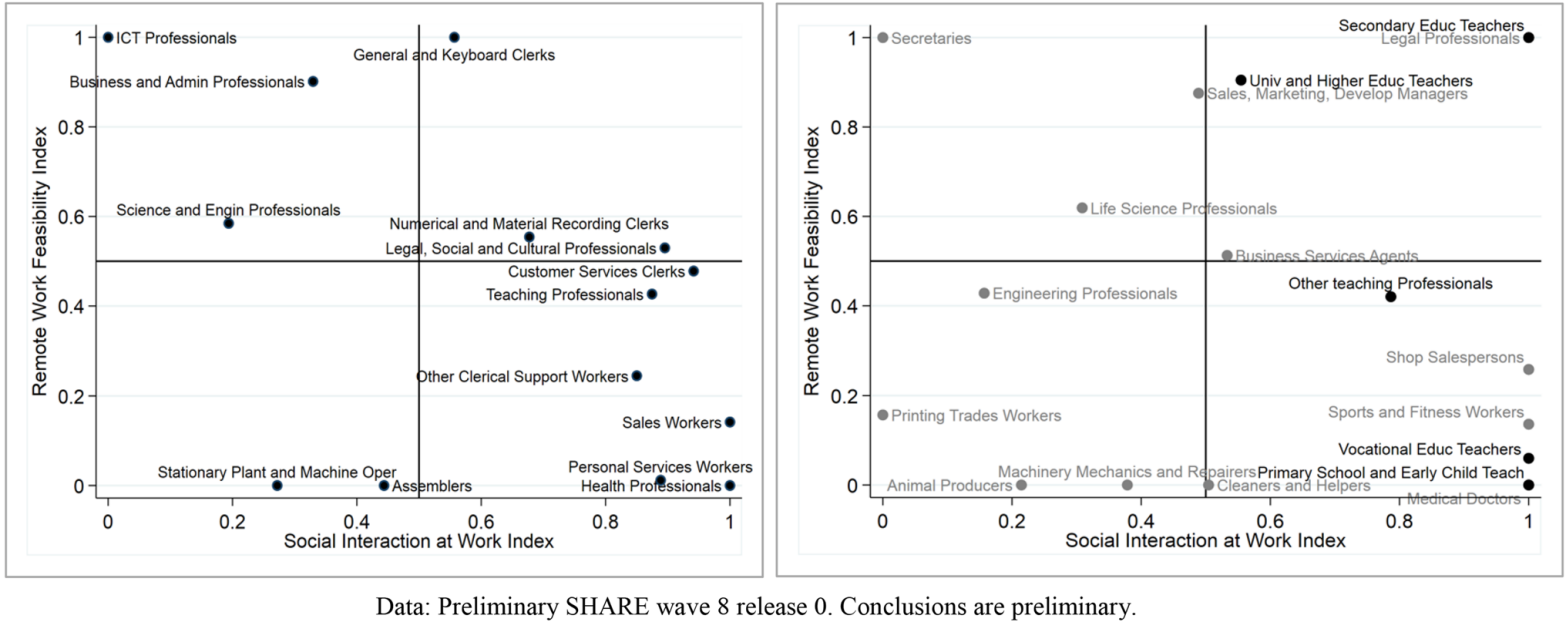
Remote work feasibility and social interaction indexes on a selection of ISCO-08 2-digit codes (left panel), and ISCO-08 3-digit codes (right panel)

Table 2 displays the values of these two indexes for each ISCO-08 3-digit job. Interestingly, we do not find a defined pattern characterising these measures: while some occupations display high remote work feasibility and low social contacts (e.g. legal professionals), others are technically teleworkable but would normally require intensive social interactions (for example 233 — secondary education teachers). This is also easily observed in Fig. 3, which matches the two indexes for a selection of ISCO-08 sub-majors in the left panel and for several ISCO-08 3-digit codes in the right panel. This comparison gives an idea of the significant heterogeneity, which exists among occupations even within the same sub-major. For instance, important differences appear when looking at professional teachers (23) and their detailed decomposition by 3-digit codes. University and higher education teachers (231) display high remote work feasibility and mild social contacts, but primary school and early childhood teachers (234) on the other hand, require strong interactions with children. Furthermore, secondary school teachers (233) would normally require intensive social interactions yet have features which are well suited to home working.

**Table 2:** Remote work feasibility and social interaction indexes by ISCO-08 3-digit codes

ISCO-08 3-digit	Occupation	Remote work feasibility	Social interaction
111	Legislators and Senior Officials	0,970	0,853
112	Managing Directors and Chief Executives	1	0,912
121	Business Services and Administration Managers	0,971	0,528
122	Sales, Marketing and Development Managers	0,875	0,489
131	Production Managers in Agriculture, Forestry and Fisheries	0	0,667
132	Manufacturing, Mining, Construction and Distribution Managers	0,289	0,413
133	Information and Communications Technology Services Managers	1	0
134	Professional Services Managers	0,731	0,616
141	Hotel and Restaurant Managers	0	1
142	Retail and Wholesale Trade Managers	1,000	1
143	Other Services Managers	0,743	0,469
211	Physical and Earth Science Professionals	0,481	0,055
212	Mathematicians, Actuaries and Statisticians	1,000	0
213	Life Science Professionals	0,619	0,309
214	Engineering Professionals (excluding Electrotechnology)	0,428	0,157
215	Electrotechnology Engineers	0,824	0,000
216	Architects, Planners, Surveyors and Designers	0,672	0,459
221	Medical Doctors	0	1
222	Nursing and Midwifery Professionals	0	1
223	Traditional and Complementary Medicine Professionals	0	1
224	Paramedical Practitioners	0	1
225	Veterinarians	0	1
226	Other Health Professionals	0	1
231	University and Higher Education Teachers	0,905	0,555
232	Vocational Education Teachers	0,060	1
233	Secondary Education Teachers	1	1
234	Primary School and Early Childhood Teachers	0	1
235	Other Teaching Professionals	0,420	0,787
241	Finance Professionals	1,000	0,445
242	Administration Professionals	0,752	0,289
243	Sales, Marketing and Public Relations Professionals	1	0,214
251	Software and Applications Developers and Analysts	1	0
252	Database and Network Professionals	1	0
261	Legal Professionals	1	1
262	Librarians, Archivists and Curators	0,879	0,938
263	Social and Religious Professionals	0,084	0,937
264	Authors, Journalists and Linguists	0,796	0,439
265	Creative and Performing Artists	0,633	0,903
311	Physical and Engineering Science Technicians	0	0,240
312	Mining, Manufacturing and Construction Supervisors	0	1
313	Process Control Technicians	0	0,862
314	Life Science Technicians and Related Associate Professionals	0	0,455
315	Ship and Aircraft Controllers and Technicians	0	0,824
321	Medical and Pharmaceutical Technicians	0	1
322	Nursing and Midwifery Associate Professionals	0	1
323	Traditional and Complementary Medicine Associate Professionals	0	1
324	Veterinary Technicians and Assistants	0	1
325	Other Health Associate Professionals	0,113	0,905
331	Financial and Mathematical Associate Professionals	0,959	0,940
332	Sales and Purchasing Agents and Brokers	1	0,592
333	Business Services Agents	0,513	0,533
334	Administrative and Specialized Secretaries	0,797	0,920
335	Government regulatory associate professionals	0,364	0,886
341	Legal, Social and Religious Associate Professionals	0,496	0,917
342	Sports and Fitness Workers	0,136	1
343	Artistic, Cultural and Culinary Associate Professionals	0,073	0,974
351	Information and Communications Technology Operations and User Support Technicians	1	0,577
352	Telecommunications and Broadcasting Technicians	0,078	0,597
411	General Office Clerks	1	0,978
412	Secretaries (general)	1	0
413	Keyboard Operators	1	0,233
421	Tellers, Money Collectors and Related Clerks	0,833	0,737
422	Client Information Workers	0,403	0,989
431	Numerical Clerks	1	0,860
432	Material recording and Transport Clerks	0,101	0,492
441	Other Clerical Support Workers	0,242	0,848
511	Travel Attendants, Conductors and Guides	0,092	1
512	Cooks	0	1
513	Waiters and Bartenders	0	1
514	Hairdressers, Beauticians and Related Workers	0,032	1
515	Building and Housekeeping Supervisors	0	0,177
516	Other Personal Services Workers	0,047	1
521	Street and Market Salespersons	0,023	1
522	Shop Salespersons	0,258	1
523	Cashiers and Ticket Clerks	0	1
524	Other Sales Workers	0,083	1
531	Child Care Workers and Teachers’ Aides	0,008	1
532	Personal Care Workers in Health Services	0,005	1
541	Protective Services Workers	0	1
611	Market Gardeners and Crop Growers	0	0,488
612	Animal Producers	0	0,215
613	Mixed Crop and Animal Producers	0	0,079
621	Forestry and Related Workers	0	0,282
622	Fishery Workers, Hunters and Trappers	0	0,215
631	Subsistence Crop Farmers	0	0,500
632	Subsistence Livestock Farmers	0	1
633	Subsistence Mixed Crop and Livestock Farmers	0	0,558
711	Building Frame and Related Trades Workers	0	1
712	Building Finishers and Related Trades Workers	0	0,848
713	Painters, Building Structure Cleaners and Related Trades Workers	0	0,823
721	Sheet and Structural Metal Workers, Moulders and Welders, and Related Workers	0	0,416
722	Blacksmiths, Toolmakers and Related Trades Workers	0	0,034
723	Machinery Mechanics and Repairers	0	0,379
731	Handicraft Workers	0,037	0,515
732	Printing Trades Workers	0,157	0
741	Electrical Equipment Installers and Repairers	0	0,968
742	Electronics and Telecommunications Installers and Repairers	0	0,991
751	Food Processing and Related Trades Workers	0	0,588
752	Wood Treaters, Cabinet-makers and Related Trades Workers	0	0,510
753	Garment and Related Trades Workers	0	0,426
754	Other Craft and Related Workers	0	1
811	Mining and Mineral Processing Plant Operators	0	0,380
812	Metal Processing and Finishing Plant Operators	0	0,338
813	Chemical and Photographic Products Plant and Machine Operators	0	0,157
814	Rubber, Plastic and Paper Products Machine Operators	0	0,307
815	Textile, Fur and Leather Products Machine Operators	0	0,589
816	Food and Related Products Machine Operators	0	0,266
817	Wood Processing and Papermaking Plant Operators	0	0
818	Other Stationary Plant and Machine Operators	0	0,046
821	Assemblers	0	0,444
831	Locomotive Engine Drivers and Related Workers	0	0,451
832	Car, Van and Motorcycle Drivers	0	1
833	Heavy Truck and Bus Drivers	0	1
834	Mobile Plant Operators	0	0,165
835	Ships’ Deck Crews and Related Workers	0	1
911	Domestic, Hotel and Office Cleaners and Helpers	0	0,504
912	Vehicle, Window, Laundry and Other Hand Cleaning Workers	0	0,028
921	Agricultural, Forestry and Fishery Labourers	0	0,953
931	Mining and Construction Labourers	0	0,992
932	Manufacturing Labourers	0	0,715
933	Transport and Storage Labourers	0	0,869
941	Food Preparation Assistants	0	1
952	Street Vendors (excluding Food)	1	1
961	Refuse Workers	0	1
962	Other Elementary Workers	0,010	0,949

All these examples reveal the importance of using detailed job information in order to better understand the role of job characteristics during the first wave of the Pandemic.

## Empirical strategy

Our paper enquires about individuals’ working experiences during the Pandemic: more specifically having undergone work interruption spells and the length of such episodes. We perform the analysis in two steps: first, we estimate the effect of occupation on the probability of having experienced work interruptions (temporary or permanent) using a probit regression; second, we analyse the correlation between the job features and the length of such spells by running an ordered probit specification.

A simple regression model for both outcomes is given in Eq. (1):

1 $$\begin{gathered} y_{i} = \alpha + \beta _{1} \;Essential_{i} + \beta _{2} \;\text{Re} moteWorkIndex_{i} \hfill \\ \;\;\;\;\;\; + \beta _{3} \;SocialInter\_Index_{{\text{i}}} + \beta _{4} \;Essential_{{\text{i}}} *\text{Re} moteWorkIndex_{{\text{i}}} \hfill \\ \;\;\;\;\;\; + \beta _{5} \;Essential_{{\text{i}}} *SocialInter\_Index_{{\text{i}}} + X'_{{\text{i}}} + \rho _{{\text{c}}} + u_{{\text{i}}} \hfill \\ \end{gathered}$$

In the first specification, the dependent variable ${y}_{i}$ is a binary variable taking the value of 1 if the respondent has experienced work interruptions and 0 otherwise. When estimating the length of work breaks we define ${y}_{i}$ as a categorical ordered variable based on the number of weeks of interruption reported by the respondent. In this case, the dependent variable ${y}_{i}$ takes value of 0 if no interruptions were declared, value of 1 if the respondent stopped his/her working activity for at most 8 weeks and a value of 2 if the interruption lasted for more than 8 weeks. The key explanatory variables are the occupation specific variables: the two indexes measuring the remote work feasibility ${(RemoteWorkIndex}_{i})$ and the intensity of social interaction $(SocialInter\_Index_{i} )$, as well as an indicator variable that identifies the essential occupations (${Essential}_{i}=1$). The use of pre-COVID-19 questions about job characteristics enables us to overcome possible endogeneity concerns between job features and work outcomes during the Pandemic.

In all the specifications we control also for other determinants concerning workers and work environment. A particularly relevant variable is the self-evaluated IT skills of the individual—which is recovered from the previous waves of SHARE. We also control for a set of socioeconomic and demographic variables, such as gender, age, education, health status (whether the individual experienced major illnesses immediately before the Pandemic), whether the individual used to work as a private employee, public sector employee or was self-employed. Moreover, in order to account for heterogeneities among countries—both in terms of lockdown measures as well as economic background—we include country fixed-effect dummy variables (${{\varvec{\rho}}}_{{\varvec{c}}}$).

## Results

### Occupation, work interruption and work arrangements during the Pandemic

Table 3 reports the marginal effects of the probability of work interruptions for two specifications: the first column—model 1—is a parsimonious specification in which, besides the occupation specific variables (i.e. the essential nature of a job, the remote work feasibility index and the social interaction index), we include gender, age and country of residence. In model 2, we also control for education, information technology skills, type of employment and health status.

**Table 3:** Probit model: work interruption probability

	Work Interruption	
	Baseline model	Full model
Essential Jobs	− 0.061***	− 0.035***
	(0.009)	(0.01)
Remote Work Feasibility Index	− 0.119***	− 0.078***
	(0.012)	(0.014)
Social Interaction Index	0.009	0.036**
	(0.014)	(0.015)
Age	0.001	0.0003
	(0.001)	(0.001)
Female	0.023***	0.039***
	(0.009)	(0.009)
*High School Education (baseline)*		
Less than high school	−	0.030**
		(0.016)
Higher than high school	−	− 0.035***
		(0.011)
Major Illness	−	0.02
		(0.017)
*Medium level of IT− skills (baseline)*		
High IT− skills	−	− 0.005
		(0.011)
Low IT− skills	−	0.007
		(0.013)
Private Employee (*baseline*)		
Public employee	−	− 0.083***
		(0.01)
Self− employed	−	0.064***
		(0.016)
Essential_RemoteWorkIndex	Yes	Yes
Essential_SocialInteractionIndex	Yes	Yes
Country dummies	Yes	Yes
N	7619	6878
Pseudo− r2	0.0925	0.1086
Log pseudolikelihood	− 3246.16	− 2910.38

Data: Preliminary SHARE wave 8 release 0. Conclusions are preliminary.

Note: Average marginal effects of probit models are reported. Drop of observations in full model is due to missing values in additional explanatory variables.

*p < 0.1, **p < 0.05, ***p < 0.01

**Fig. 4:**
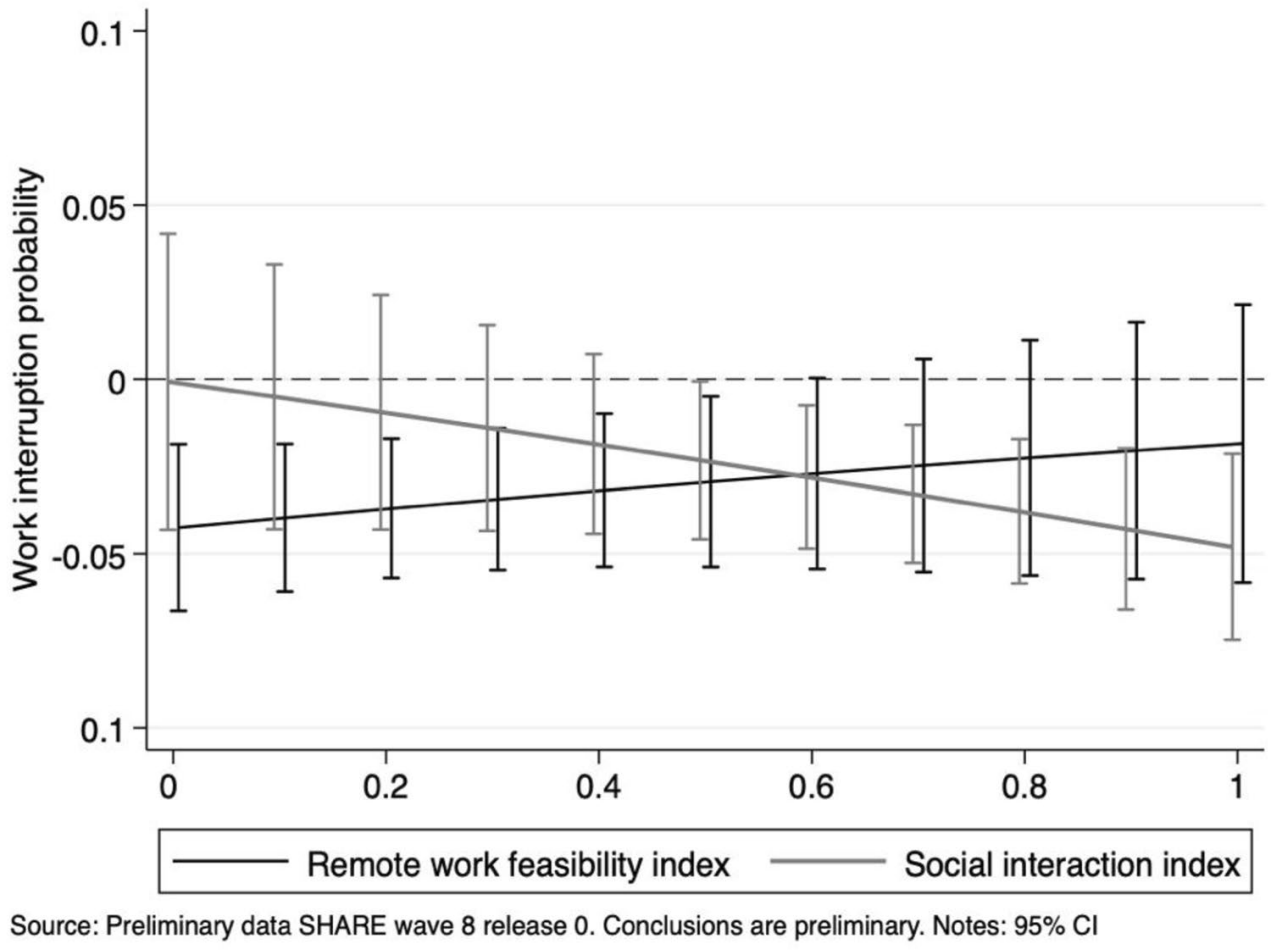
Work interruption probability: average marginal effects of working in essential jobs at different levels of the remote work feasibility and social interaction indexes

Our results point out job characteristics as major determinants of the probability to experience work interruptions during the first—unexpected—wave of the Pandemic. Indeed, individuals employed in “essential” activities were 3.5 percentage points less likely to have gone through work breaks than those working in “unessential” jobs. In relative terms, with respect to the average sample probability of 17.9%, individuals employed in essential jobs were 19.5% less likely to experience interruptions with respect to non-essential employees. The marginal effects of the two indexes reveal that increasing suitability of remote work is associated with a significantly lower probability of work interruptions, while the higher the level of social interaction in the workplace, the larger the likelihood of experiencing work breaks.^11^ Figure 4 provides additional insights by displaying the marginal effects of an essential job at various levels of remote work feasibility index and social interaction index.

It is worth noticing that, for values of the remote working feasibility index smaller than 0.6 (representing little or modest home working suitability), being an essential occupation is associated with significantly lower probabilities of experiencing work interruptions. The positive slope suggests that, as the technical teleworkability of a job increases, the gap between essential and unessential occupations gets smaller with respect to the likelihood of work breaks during the Pandemic. An opposite relationship is found when looking at the social interaction index: jobs characterized by a low intensity of interaction between people display no significant differences between essential and unessential activities. On the contrary, as the level of social contact becomes more important, the difference between the two categories increases (i.e. essential jobs display lower probabilities of work interruption).

One could argue that the three selected job characteristics (i.e. essential/unessential plus the two indexes) might be arbitrary and conceal useful information because they are based on criteria reflecting the COVID-19 Pandemic. Indeed, as the Pandemic occurred, some jobs became more relevant than others and at the same time, some occupations were more prone to home working or less risky in terms of social interaction intensity. In order to show that our proposed measures preserve the value of the original information, we carried out a robustness check (see Table 4) by estimating Eq. (1) with forty dummy variables, one for each job sub-major. Note that in this setup sometimes we treat in a unique group rather heterogeneous occupations, due to how the ISCO-08 2-digit classification clusters jobs. For example, we cannot distinguish between sellers of food (“essential” goods) and those vending other commodities.^12^

**Table 4:** Work Interruption probability by ISCO-08 2-digits

	Baseline model	Full model	(continue)	Baseline model	Full model
*23.Teaching professionals (BASELINE GROUP)*					
11.Chief Executives, Senior Officials and Legislators	0.066**	0.036			
	(0.030)	(0.032)			
12.Administrative and Commercial Managers	0.071**	0.038	53.Personal Care Workers	0.121***	0.096***
	(0.028)	(0.031)		(0.024)	(0.028)
13.Production and Specialized Services Managers	0.041	0.013	54.Protective Services Workers	0.123**	0.103**
	(0.026)	(0.029)		(0.038)	(0.042)
14.Hospitality, Retail and Other Services Managers	0.294***	0.230***	61.Market-oriented Skilled Agricultural Workers	0.018	− 0.040
	(0.052)	(0.053)		(0.025)	(0.026)
21.Science and Engineering Professionals	0.050**	0.012	62.Market-oriented Skilled Forestry, Fishery and Hunting.	0.032	− 0.044
	(0.022)	(0.025)		(0.068)	(0.063)
22.Health Professionals	0.016	0.000	63.Subsistence Farmers, Fishers, Hunters and Gatherers	− 0.014	− 0.055
	(0.017)	(0.021)		(0.039)	(0.038)
24.Business and Administration Professionals	0.014	− 0.011	71.Building and Related Trades Workers (excluding Electr..)	0.134***	0.063**
	(0.021)	(0.024)		(0.029)	(0.031)
25.Information and Communications Technology Professionals	0.093**	0.072	72.Metal, Machinery and Related Trades Workers	0.194***	0.147***
	(0.042)	(0.047)		(0.030)	(0.034)
26.Legal, Social and Cultural Professionals	0.065***	0.046*	73.Handicraft and Printing Workers	0.275***	0.233***
	(0.021)	(0.025)		(0.057)	(0.061)
31.Science and Engineering Associate Professionals	0.116***	0.091***	74.Electrical and Electronic Trades Workers	0.142***	0.116***
	(0.027)	(0.030)		(0.042)	(0.044)
32.Health Associate Professionals	0.110***	0.093***	75.Food Processing, Woodworking, Garment and Other Craft.	0.170***	0.116***
	(0.030)	(0.034)		(0.031)	(0.034)
33.Business and Administration Associate Professionals	0.073***	0.049**	81.Stationary Plant and Machine Operators	0.129***	0.092**
	(0.018)	(0.022)		(0.035)	(0.038)
34.Legal, Social, Cultural and Related Associate Professi.	0.115**	0.091**	82.Assemblers	0.141**	0.104
	(0.036)	(0.041)		(0.063)	(0.065)
35.Information and Communications Technicians	0.166***	0.135**	83.Drivers and Mobile Plant Operators	0.182***	0.144***
	(0.064)	(0.064)		(0.027)	(0.031)
41.General and Keyboard Clerks	0.037**	0.015	91.Cleaners and Helpers	0.160***	0.122***
	(0.019)	(0.023)		(0.025)	(0.029)
42.Customer Services Clerks	0.108***	0.090**	92.Agricultural, Forestry and Fishery Labourers	0.141**	0.061
	(0.035)	(0.039)		(0.066)	(0.059)
43.Numerical and Material Recording Clerks	0.089***	0.057*	93.Labourers in Mining, Construction, Manufacturing and T.	0.152***	0.111***
	(0.027)	(0.030)		(0.031)	(0.034)
44.Other Clerical Support Workers	0.061*	0.057	94.Food Preparation Assistants	0.341***	0.327***
	(0.031)	(0.038)		(0.065)	(0.070)
51.Personal Service workers	0.295***	0.243***	95.Street and Related Sales and Services Workers	0.389**	0.223
	(0.027)	(0.030)		(0.175)	(0.179)
52.Sales Workers	0.180***	0.130***	96.Refuse Workers and Other Elementary Workers	0.064*	0.039
	(0.024)	(0.027)		(0.038)	(0.044)

Data: Preliminary SHARE wave 8 release 0. Conclusions are preliminary.

Notes: Average marginal effects of probit models are reported. Baseline model: 7619 (observations), 0.121 (Pseudo R2), − 3143,11 (Log pseudolikelihood). Full model: 6878 (observations), 0.1316 (Pseudo R2), − 2835.22 (Log pseudolikelihood)

*p < 0.1, **p < 0.05, ***p < 0.01

We choose “teaching professionals” as the baseline group due to their fairly homogeneous nature in terms of work arrangements options during the Pandemic: most teaching activities continued remotely in almost every European country. With respect to the baseline group, the coefficients show that jobs belonging to other sub-majors had significantly higher probabilities of temporary or permanent work interruptions. Larger and statistically significant effects are associated with occupations related to tourism and hospitality, while jobs in “subsistence agricultural activities” were found to have a lower probability of interruptions. These results are in line with our main specifications.

Table 5 reports the marginal effects of two ordered probit models for the length of work interruptions. Individuals working in essential occupations were about 1.3–1.6 percentage points less likely to experience longer work interruptions (columns 2 and 3 respectively) and more likely to go through brief episodes (less than 1 week) or no activity stop (column 1 of each specification), with respect to the “unessential” ones. In relative terms, being employed in an essential activity determines a reduction in the probability of a brief or long interruption of about 12.15% and 22.22%, respectively. Instead, the magnitude of the effect when considering the probability of zero weeks of interruption is much smaller, i.e. no interruptions at all (+ 3.53%). Jobs with high suitability to remote work display significantly lower probabilities of longer work breaks, while those with a large intensity of social interactions have higher likelihoods of prolonged interruptions. The results are consistent with those found in the estimation of the probability of stopping work. As a robustness check, we also perform a Tobit regression model using the number of weeks of interruption as a continuous dependent variable. The results support our findings and are available as supplementary information.

**Table 5:** Ordered probit model: length of work interruption

	Length of work interruption			Length of work interruption		
	0 weeks	weeks	> 8 weeks	0 weeks	1–8 weeks	> 8 weeks
Essential Jobs	0.057***	− 0.027***	− 0.029***	0.029***	− 0.013***	− 0.016***
	(0.009)	(0.004)	(0.004)	(0.010)	(0.005)	(0.005)
Remote work feasibility index	0.113***	− 0.054***	− 0.059***	0.074***	− 0.034***	− 0.039***
	(0.012)	(0.006)	(0.006)	(0.013)	(0.006)	(0.007)
Social interaction index	− 0.012	0.005	0.007	− 0.037**	0.017**	0.020***
	(0.013)	(0.006)	(0.007)	(0.014)	(0.007)	(0.008)
Age	− 0.001	0,001	0.001	− 0.001	0.0003	0.0003
	(0.001)	(0.0004)	(0.0004)	(0.001)	(0.0005)	(0.001)
Female	− 0.023***	0.011***	0.012***	− 0.039***	0.018***	0.021***
	(0.009)	(0.004)	(0.005)	(0.009)	(0.004)	(0.005)
*High School Education (baseline)*						
Less than high school	−	−	−	− 0.025*	0.011*	0.014*
	−	−	−	(0.015)	(0.007)	(0.008)
Higher than high school	−	−	−	0.038***	− 0.018***	− 0.020***
	−	−	−	(0.010)	(0.005)	(0.005)
Major illness	−	−	−	− 0.029*	0.013*	0.016
	−	−	−	(0.017)	(0.008)	(0.010)
*Medium level of IT− skills (baseline)*						
High IT− skills	−	−	−	0.004	− 0.002	− 0.002
	−	−	−	(0.011)	(0.005)	(0.006)
Low IT− skills	−	−	−	− 0.001	0.0003	0.0004
	−	−	−	(0.012)	(0.006)	(0.007)
Private Employee (*baseline*)	−	−	−			
Public employee	−	−	−	0.083***	− 0.042***	− 0.041***
	−	−	−	(0.010)	(0.005)	(0.005)
Self− employed	−	−	−	− 0.060***	0.025***	0.034***
	−	−	−	(0.015)	(0.006)	(0.009)
Essential_RemoteWorkIndex	Yes			Yes		
Essential_SocialInteractionIndex	Yes			Yes		
Country dummies	Yes			Yes		
N	7619			6878		
Pseudo− r2	0.0724			0.0852		
Log pseudolikelihood	− 4169.13			− 3760.93		

Data: Preliminary SHARE wave 8 release 0. Conclusions are preliminary. Notes: Average marginal effects of probit models are reported. Drop of observations in full model due to missing values in additional explanatory variables

*p < 0.1, **p < 0.05, ***p < 0.01

**Fig. 5:**
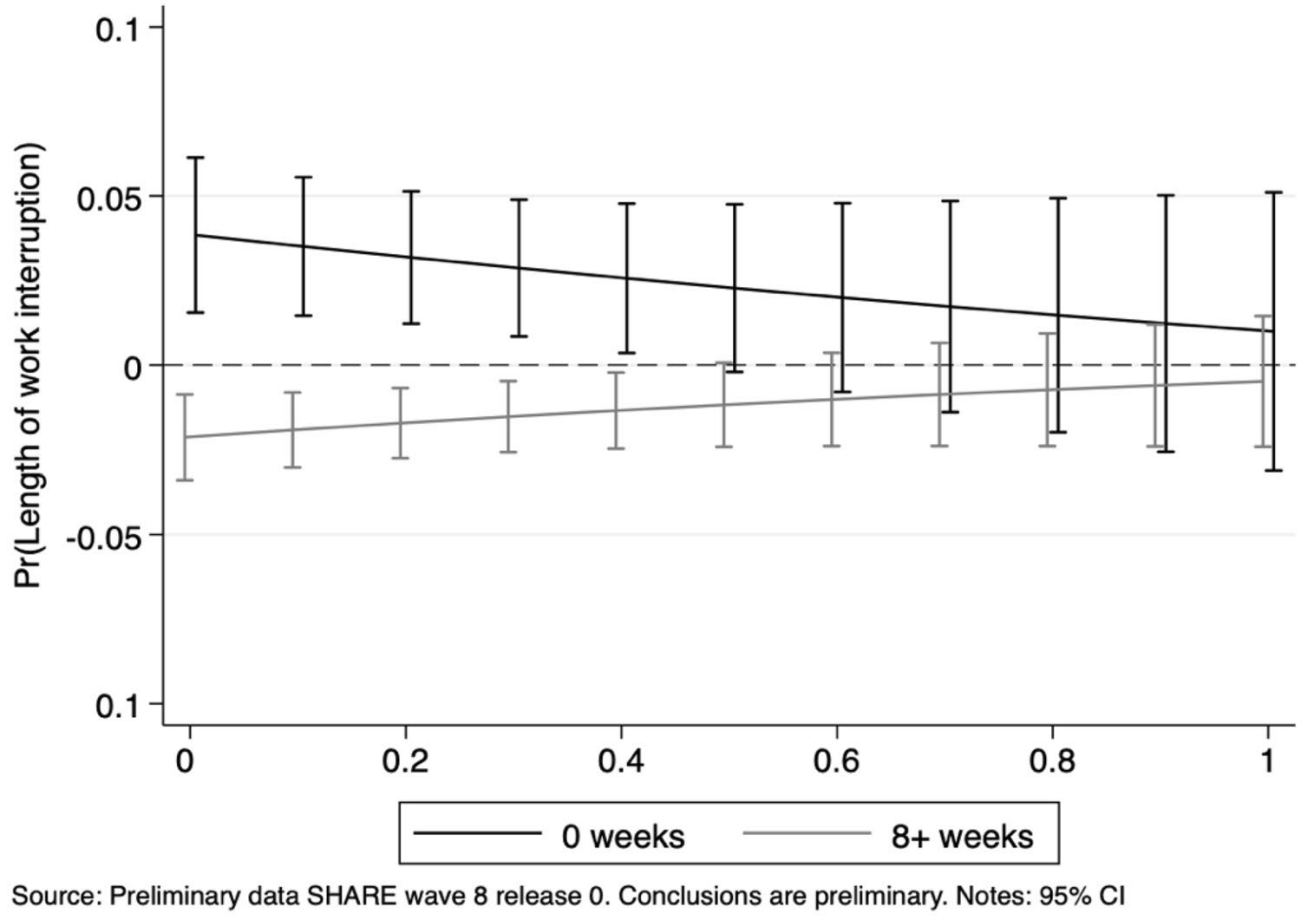
Length of work interruption: average marginal effects of working in essential jobs at different level of the remote work feasibility index

**Fig. 6:**
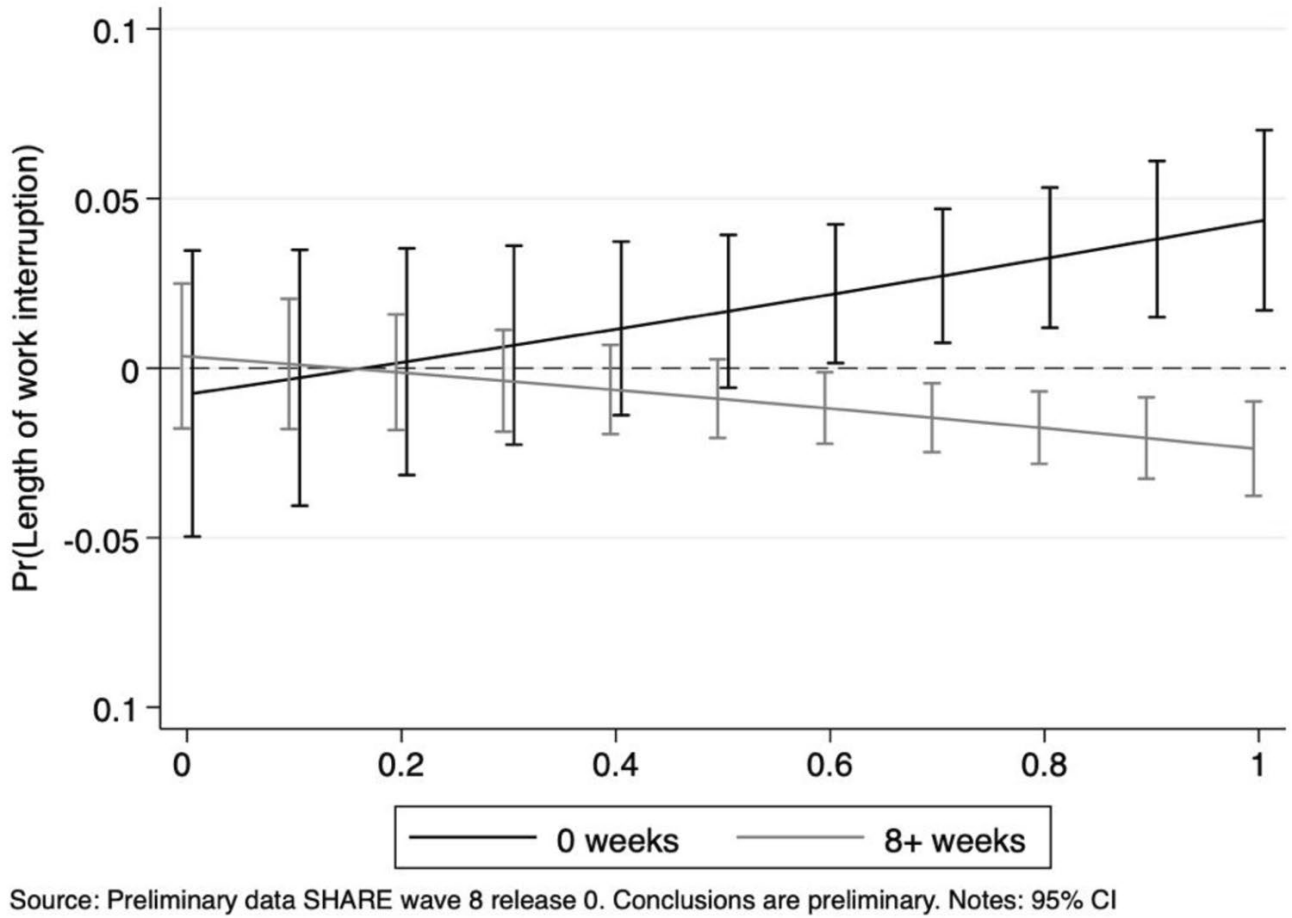
Length of work interruption: average marginal effects of working in essential jobs at different level of the social interaction index

Additional insights into the impact of job features and their magnitude are provided by Figs. 5 and 6: they show the average marginal effects of an essential occupation on the probability of having experienced 0, 1 to 8 and more than 8 weeks of work interruption at different values of the two indexes. Workers employed in “essential” occupations unsuited to remote work (index values smaller than 0.5) display a significantly lower probability of having prolonged work interruptions with respect to workers performing “unessential” jobs. Such a difference vanishes as home work feasibility increases. A similar impact—but opposite in sign—is observed for the social interaction index: jobs characterised by intensive social contacts but regarded as “crucial” in society, reveal a reduction in the likelihood of experiencing longer work interruptions with respect to non-essential ones. This effect disappears at lower values of the social interaction index.

In addition to the previous finding, our results add salient evidence on several other issues. We find that education has a clear mitigating role for the negative labour market effects of the Pandemic, even when controlling for occupation features. Respondents holding higher levels of education (vis-a-vis the reference category “high school degree”) display a 3.5 percentage points lower likelihood of work interruption and about 2 percentage points smaller probability of undergoing prolonged interruption spells. We speculate that educational attainment plays a relevant role per se, both because workers with higher education are often associated with “higher quality jobs”, and also because education is related to the specific tasks required in a job. The idea is that the human capital of highly educated workers may be more flexible in terms of tasks performed. By recalling the basic characteristics defined by Autor and Dorn, 2009, Autor and Dorn 2013 and Deming 2017, the exogenous shock generated by the Pandemic has probably affected more jobs involving tasks of high *routine intensity*, i.e. tasks that involve a well-defined repetitive set of procedures. In fact, during economic downturns, sizeable employment losses mainly appear among the more routine-intensive middle-skilled occupations, some of these jobs eventually disappear and are not retrieved when the economy recovers (Jaimovich and Siu, 2020).

Finally, our results also highlight differences between workers in different types of employment. With respect to the baseline category of the private employees, public employees were 8.3 percentage points less likely to have experienced work interruption whereas self-employed workers had significantly larger probabilities of such an event. Moreover, public sector employees are characterised by a 4.1 percentage points lower probability of having experienced work interruptions between 1 and 8 weeks, and 4.2 percentage points smaller likelihood of breaks longer than 8 weeks. We find an opposite and significant effect for self-employed workers.

### A focus on women

The previous models allow us to address several questions that are currently the object of debate for researchers and policy makers. Did women pay a higher price than men in terms of work interruptions during the Pandemic? Are there heterogeneities in terms of job characteristics useful to build more targeted (and potentially more effective) support measures?

By recalling that particular care should be paid in drawing general conclusions—our sample looks at workers aged 50 and over—we attempt to provide answers to the above questions. When introducing a “female dummy” in the above models, we find that women in our age groups are more likely to experience work interruptions with respect to men (about 3.9 percentage points more), and longer work breaks (by 1.8 pp more for interruptions between 1 to 8 weeks, and by 2.1 pp more for episodes longer than 8 weeks). In relative terms, women have been 21.79% more likely to experience working breaks than men. Moreover, by looking at the duration of such interruptions, they also display higher probabilities of short and long breaks of about + 16.5% and + 29.16%, respectively. In order to get further insights, we run the regressions separately by gender. Table 6 reports the results of these estimations both for the probability and for the length of work interruptions.

**Table 6:** Probability of a work interruption and its length by gender

	Work Interruption		Length of work interruption					
			0 weeks		1 to 8 weeks		More than 8 weeks	
	Women	Men	Women	Men	Women	Men	Women	Men
Essential Jobs	0.057***	− 0.008	0.051***	0.006	− 0.023***	− 0.003	− 0.027***	− 0.003
	(0.014)	(0.015)	(0.014)	(0.015)	(0.007)	(0.007)	(0.007)	(0.008)
Remote work feasibility index	− 0.085***	− 0.057***	0.083***	0.049**	− 0.038***	− 0.023**	− 0.045***	− 0.026**
	(0.019)	(0.020)	(0.018)	(0.020)	(0.009)	(0.009)	(0.010)	(0.010)
Social interaction index	0.034	0.045**	− 0.037*	− 0.044**	0.016	0.021**	0.022*	0.023**
	(0.021)	(0.021)	(0.021)	(0.020)	(0.010)	(0.010)	(0.011)	(0.011)
Essential_RemoteWorkIndex	Yes	Yes	Yes	Yes	Yes	Yes	Yes	Yes
Essential_SocialInteractionIndex	Yes	Yes	Yes	Yes	Yes	Yes	Yes	Yes
Country dummies	Yes	Yes	Yes	Yes	Yes	Yes	Yes	Yes
Additional covariates	Yes	Yes	Yes	Yes	Yes	Yes	Yes	Yes
N	3839	3039	3839	3039	3839	3039	3839	3039
Pseudo− r2	0.1328	0.1109	0.1049	0.0839	0.1049	0.0839	0.1049	0.0839
Log pseudolikelihood	− 1595.32	− 1266.96	− 2077.20	− 1640.01	− 2077.20	− 1640.01	− 2077.20	− 1640.01

Data: Preliminary SHARE wave 8 release 0. Conclusions are preliminary.

Notes: Average marginal effects of probit and ordered probit full models are reported. Variables “Essential_RemoteWorkIndex” and “Essential_SocialInteractionIndex” are the interaction terms.

*p<0.1, **p<0.05, ***p<0.01

**Fig. 7:**
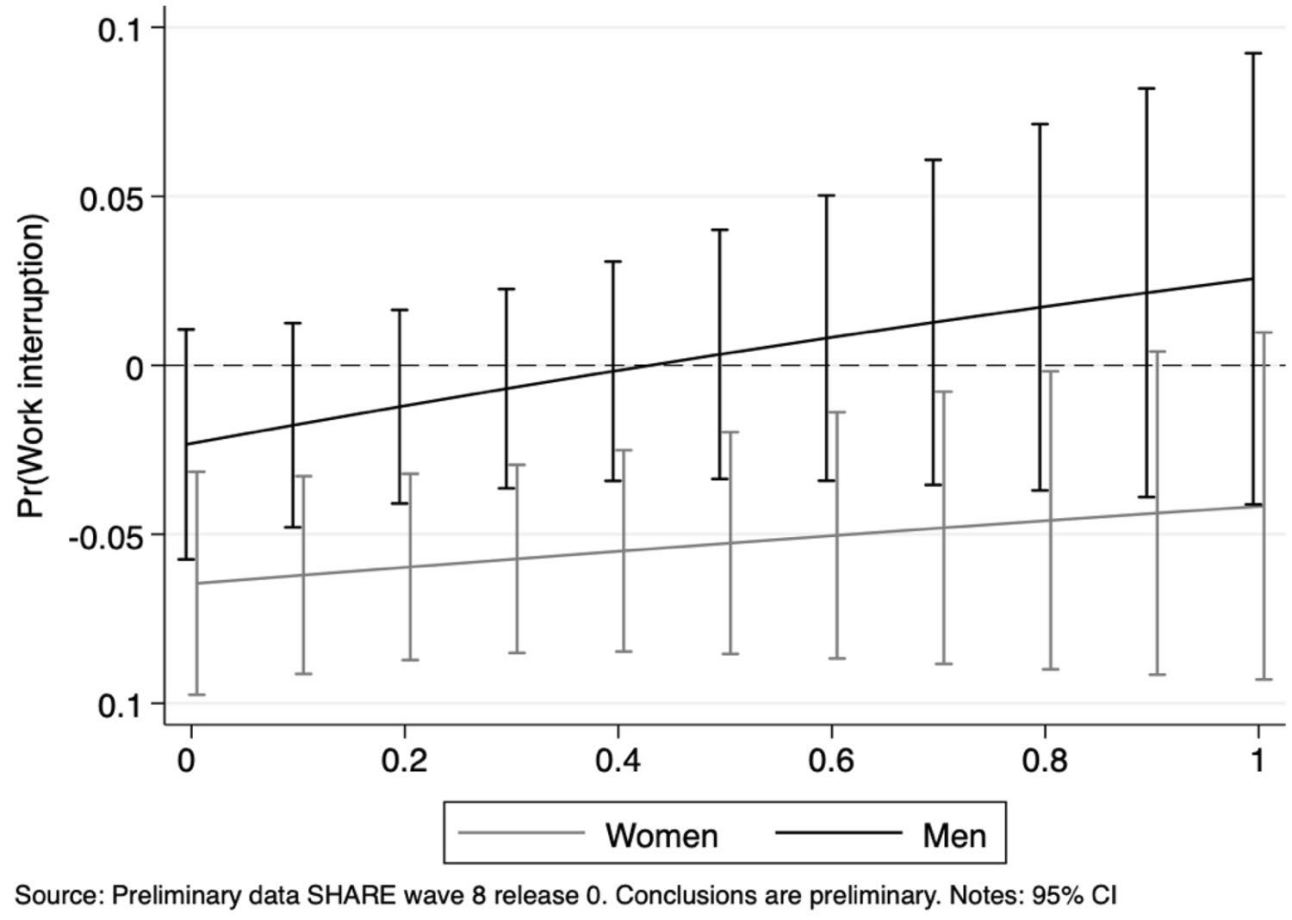
Work interruption probability by gender: average marginal effects of working in essential jobs at different levels of the remote work feasibility index

**Fig. 8:**
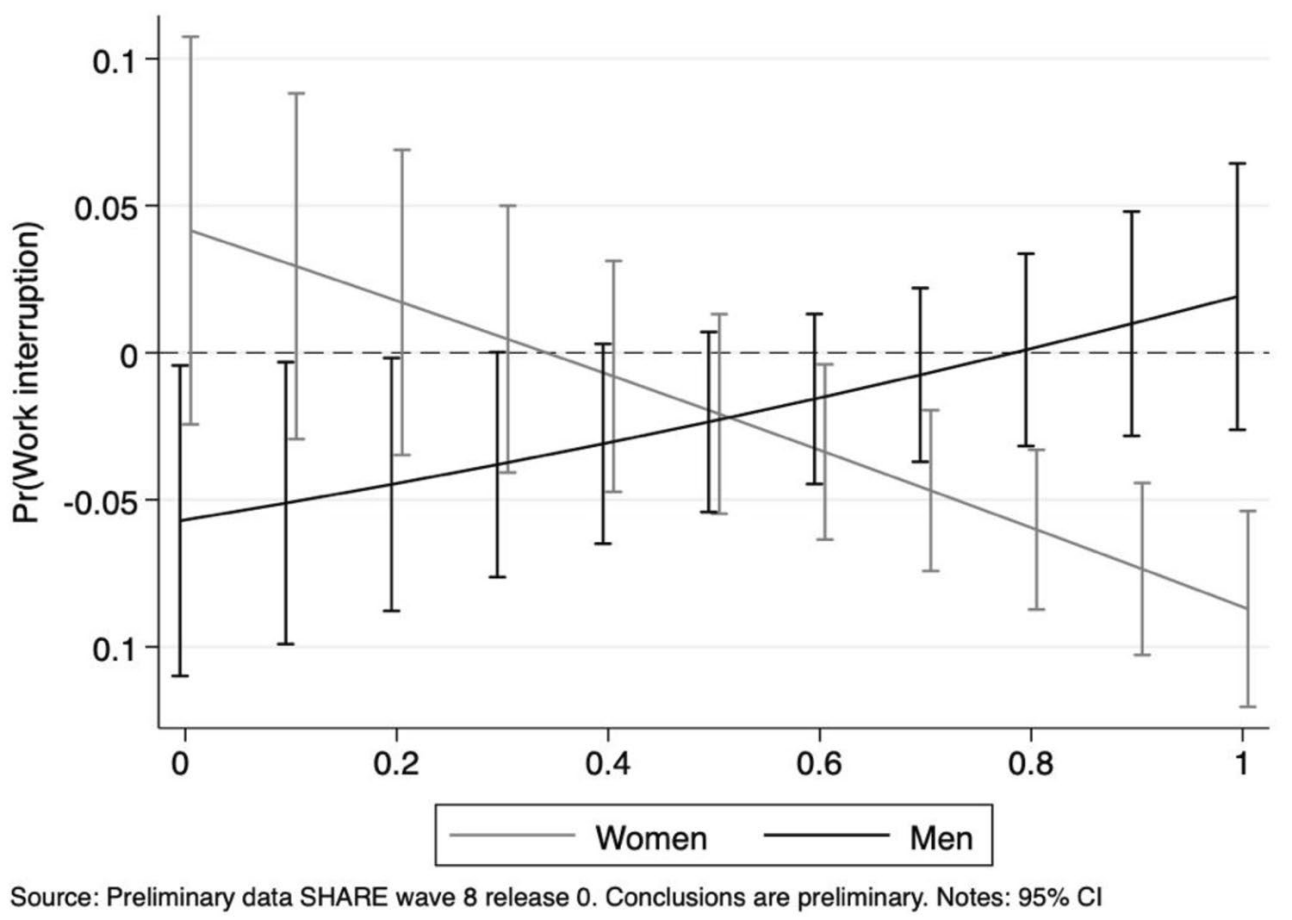
Work interruption probability by gender: average marginal effects of working in essential jobs at different levels of the social interaction index

**Fig. 9:**
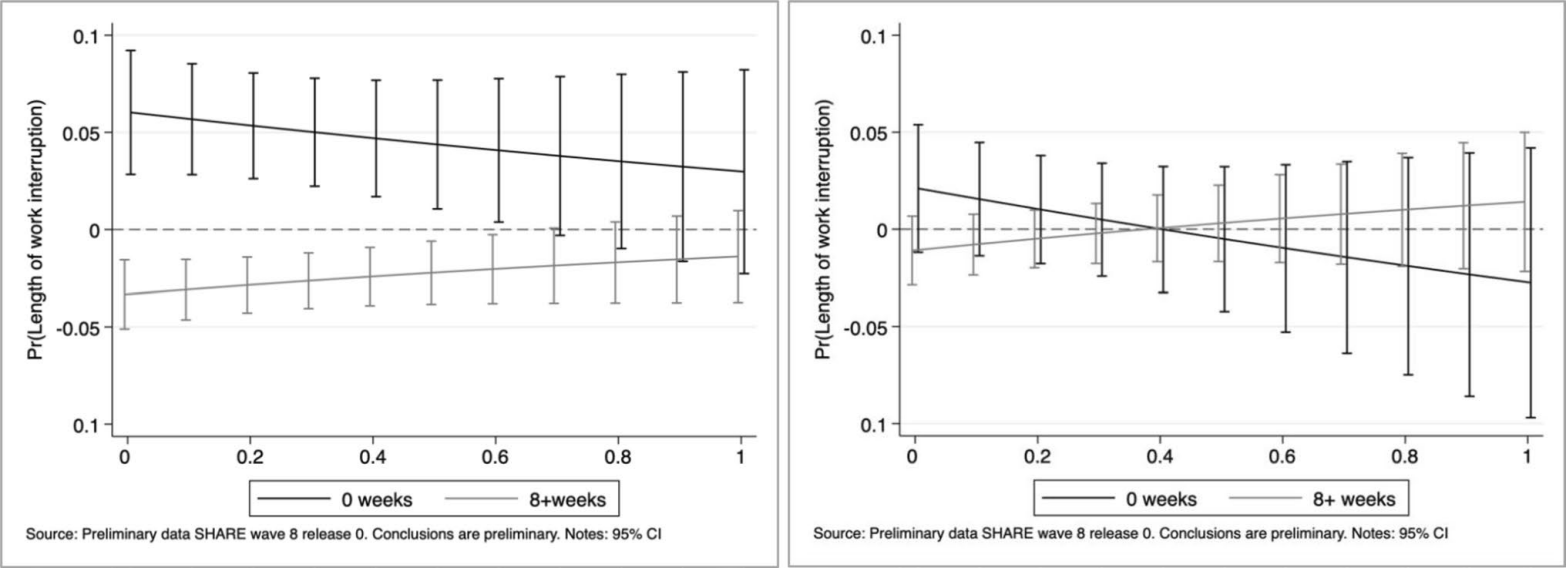
Length of work interruption by gender: average marginal effects of working in essential jobs at different levels of the remote work feasibility index for women (*left panel*) and men (*right panel*)

**Fig. 10:**
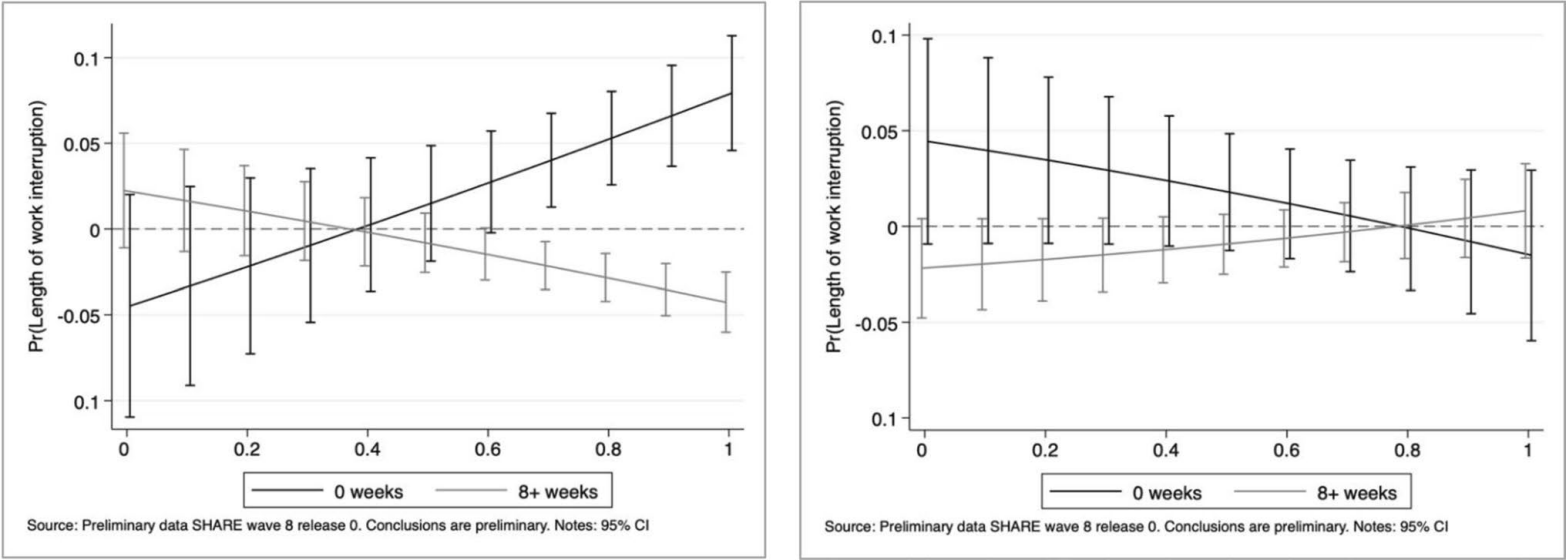
Length of work interruption by gender: average marginal effects of working in essential jobs at different levels of the social interaction index for women (*left panel*) and men (*right panel*)

It is easy to observe that a large part of the effect captured in all the main specifications by the essential nature of a job is mostly driven by women. Female workers employed in essential activities are 5.7 percentage points less likely to experience interruptions than those employed in non-essential ones. Differently, male workers seem more vulnerable as the level of social interaction at the usual workplace increases. As expected, the remote work feasibility of a job has been a crucial determinant during the first wave of the COVID-19 Pandemic, irrespective of gender. Figs 7, 8, 9, 10 depict the average marginal effects of being employed in essential occupations (with respect to non-essential ones) at different levels of remote work feasibility and social interaction, for men and women separately. The first type of interaction points out the home work feasibility as the prevailing dimension among women: at lower levels of the remote work feasibility index the essentiality of tasks performed by women is highly significant to avoid work interruptions as well as longer breaks, while this is not the case for men. As regards our second index, for high levels of social interaction at work, the essential nature of an occupation represents a deterrent against job interruption mainly for women, while the opposite is found for men.

Several aspects such as *gender* and *age composition* of specific jobs can partially explain the previous results. For instance, the prevalence of women tends to be higher among essential but more exposed to contagion (intensive social interaction) activities (i.e. nursing and midwifery professionals (222) or primary school and early childhood teachers (234)), while male workers prevail among essential but lower risk occupations (i.e. heavy truck and bus drivers (833) or mixed crop and animal producers (613)). No less important is the role played by intergenerational differences and thus, the representativeness of our sample of 50 + workers: the gender selection into specific jobs—more or less demanding in terms of tasks—might be highly pronounced among older cohorts compared to younger ones.

Overall, the previous findings show that the negative effects of the Pandemic on workers were harsher on women. However, the results also reveal that gender differences in labour market outcomes are driven by the intrinsic characteristics of the jobs/occupations they are involved in.

## Conclusions

This paper evaluates the impact of job characteristics on an important labour market issue, which emerged during the COVID-19 Pandemic: the probability of having experienced work interruptions, coupled with the length of such interruptions. Assessing the determinants of these labour market outcomes is of great policy relevance as suitable interventions can be designed to prevent important economic consequences at individual level and welfare losses for the European society at large. The key finding of our research effort is that job characteristics play a major role for workers aged 50 and over in Europe, even when controlling for other relevant determinants of labour supply, such as education, geographical location and the traditional demographic and “human capital” variables used in the literature.

The novelty of our paper rests on the richness of the SHARE data, which allows us to retrieve information on panel respondents before the COVID-19 outbreak and to relate such information to the reported level of activity during the lockdowns. The most salient feature of our work is the use of the newly coded occupations reported in SHARE and classified according to their ISCO-08 3-digit code. The level of detail provided by the occupational classification allows us to characterize the jobs based on several dimensions, by enabling us to generate two measures, extremely relevant under the Pandemic scenario: the suitability to remote work and the level of social interaction when performing the tasks in normal conditions. As an additional important aspect, we also distinguish between essential and unessential nature of the job. A further important feature of the SHARE data is the heterogeneity across countries, so that we benefit from the variability in labour markets arrangements/lockdowns across all SHARE countries during the Pandemic.

We find that for workers in the age group 50 and over, all the occupations dimensions considered played a major role in determining both the probability of working continuously during the Pandemic and the length of work breaks. Workers who experienced more work interruption (and longer breaks) were mainly engaged in “unessential” occupations that were either not suited to be performed remotely or involved intensive social contacts/low physical proximity. For reasonable large levels of the remote work feasibility index or small social interaction index, the difference in the likelihood of undergoing work breaks between essential and unessential jobs vanishes. A clear policy implication of our finding is that labour market arrangements should facilitate the more vulnerable jobs, devoting more resources to increasing the safety of these occupations, whenever possible.

Furthermore, non-essential occupations are characterized by longer job interruptions, possibly ending up into long-term unemployment experiences, which could jeopardize the chances for these workers to return to the labour market after the end of the crisis. Policies aimed at protecting work during the Pandemic should prioritize occupational groups which are more at risk of suffering these long-term consequences.

Our findings point to a number of possible research lines on how to improve the resilience of jobs in face of negative shocks, such as the COVID-19 Pandemic. One possible implication is that employers and institutions might need to plan a more careful organization at the workplace, paying attention to the nature of the tasks performed, so that it might be necessary to re-design the production process enlarging the notion of “risks” in performing a job.

In addition, our results contribute to an ongoing debate on gender differences in labour market outcomes. Women aged 50 and over have been more heavily affected by the Pandemic because they are more likely to experience job interruptions and for longer periods. A possible explanation supported by our data is that jobs which rely on close physical interaction with customers, such as, retail activities, accommodation or services to the person and which have been hit harder by the recent sanitary situation, are performed mainly by women. Our results help disentangling an important dilemma: on the one hand, women are more exposed to negative labour market experience, but, on the other hand, because they are more likely to work in the public sector, they are less affected by the negative COVID-19 shock (OECD, 2020b). We show that even controlling for the sector of employment, women are more likely to experience job interruptions and confirm that women represent a particularly “vulnerable group” as far as the labour market risk is concerned. So, it is possible that labour market arrangements which improve the safety of jobs —in the way we have defined them —could also help older women in enlarging the set of choices that would make it possible to keep on working.

## Supplementary Information

Below is the link to the electronic supplementary material.

Supplementary file1 (PDF 239 kb)

## Data Availability

SHARE data are available on the official SHARE-project website, www.share-project.org, upon registration.

## Footnotes

1 The employment status has been inferred from the SHARE COVID-19 Survey, question CAEP805_: “*At the time when Corona broke out, were you employed or self-employed, including working for family business*?”.

2 We have dropped individuals from Hungary and the Netherlands because some relevant information is missing.

3 We set the threshold at 8 weeks as this is the median value of the variable CAW003_.

4 Wave 8 was implemented right before the COVID-19 outbreak.

5 When respondents do not provide this type of information in wave 8 – either because they were not administered the regular questionnaire or because they had no change in their occupation since the previous interview – we recover their occupational code from the previous (most recent) wave in which they participated.

6 The list is based on the “Communication from the Commission on Guidelines concerning free movements of workers” during the first wave of the COVID-19 outbreak (https://ec.europa.eu/social/main.jsp?langId=en&catId=89&furtherNews=yes&newsId=9630) and the Dutch definition of key workers (https://www.government.nl/topics/coronavirus-covid-19).

7 We refer to Basso et al. (2020) for the detailed list of selected questions and value-thresholds.

8 This methodology maps US jobs’ characteristics, namely their technology and labour market conditions, into European jobs. This exercise entails some measurement error as long as technology differs across countries and European occupations. Thus, the results need to be interpreted as if the US occupational technology was in place for each labour market analysed (Basso et al. (2020)).

9 Hence, category 1 is a subset of category 2, while category 2 is a subset of category 3.

10 Eighteen questions are drawn from the “work context” module while nine are selected from the “work activities” section.

11 In relative terms, an increase of 10 percentage points in the remote work feasibility and social interaction indexes, determine a reduction of the probability of experiencing work interruptions of about 4.36% in the first case, while an increase of about 2.01% in the second case.

12 The only possibility to distinguish between different types of sellers (by using dummies for job categories) would be to use a much higher level of detail, such as 4-digit ISCO-08. Moreover, the distinction between those working in food shops and other types of sellers would need to go even deeper, that is, to occupation titles.
