# Supplementary material for: Occupation and working outcomes during the Coronavirus Pandemic
Source: Eur J Ageing. 2021 Oct 8;19(4):863–82. doi: 10.1007/s10433-021-00651-5 (PMC8500256; doi:10.1007/s10433-021-00651-5)
Supplement: Supplementary file 1 — Supplementary file1 (PDF 239 kb) [file 10433_2021_651_MOESM1_ESM.pdf]

Occupation and working outcomes during the Coronavirus Pandemic

Agar Brugiavini[1], Raluca E. Buia[1], Irene Simonetti[1]
[1]Department of Economics, University Ca' Foscari of Venice

Corresponding author: Raluca E. Buia
Email: elenabui@unive.it

European Journal of Ageing, SHARE COVID-19 Special Section

Supplementary Material

Table 1. Descriptive statistics

	N. Obs.	Mean	SD	Min	Max
Work interruption (*outcome 1*)	7619	0.179	0.383	0	1
Week of interruption (*outcome 2*)					
No interruptions (=0)	7619	0.821	0.383	0	1
1 to 8 weeks of interruption (=1)	7619	0.107	0.309	0	1
More than 8 weeks of interruption (=2)	7619	0.072	0.258	0	1
Age	7619	60.45	5.055	33	86
Female	7619	0.557	0.497	0	1
Level of education:					
Lower than high school	7606	0.128	0.334	0	1
High school	7606	0.417	0.493	0	1
Higher than high school	7606	0.455	0.498	0	1
Major illness	7615	0.079	0.270	0	1
IT-skills level:					
High IT-skills	6894	0.263	0.441	0	1
Medium IT-skills	6894	0.566	0.496	0	1
Low IT-skills	6894	0.170	0.376	0	1
Employee or self-employed:					
Private employee	7616	0.515	0.500	0	1
Public employee	7616	0.350	0.477	0	1
Self-employed	7616	0.136	0.342	0	1
Essential jobs	7619	0.378	0.485	0	1
Remote working feasibility index	7619	0.312	0.400	0	1
Social interaction index	7619	0.727	0.315	0	1

Data: preliminary SHARE wave 8 release 0. Conclusions are preliminary. Notes: the outcome variable 2 (weeks of interruption) is a categorical variable taking the value of 0 if respondents continued working, value 1 if they experienced a "short" interruption (between 1 and 8 weeks) or value 2 if they stopped working for more than 8 weeks.

Table 2. Number of observations by country

Country	Freq.	Percent	Cum.
Germany	536	7.04	7.04
Sweden	221	2.90	9.94
Spain	94	1.23	11.17
Italy	410	5.38	16.55
France	216	2.84	19.39
Denmark	483	6.34	25.73
Greece	333	4.37	30.10
Switzerland	284	3.73	33.82
Belgium	633	8.31	42.13
Israel	240	3.15	45.28
Czech Republic	254	3.33	48.62
Poland	572	7.51	56.12
Luxembourg	77	1.01	57.13
Portugal	122	1.60	58.73
Slovenia	230	3.02	61.75
Estonia	1,014	13.31	75.06
Croatia	222	2.91	77.98
Lithuania	338	4.44	82.41
Bulgaria	182	2.39	84.80
Cyprus	84	1.10	85.90
Finland	374	4.91	90.81
Latvia	184	2.42	93.23
Malta	103	1.35	94.58
Romania	147	1.93	96.51
Slovakia	266	3.49	100.00
Total	7,619	100.00	

Data: Preliminary SHARE wave 8 release 0. Conclusions are preliminary.

Table 3. Remote working feasibility and social interaction indexes by ISCO08 2-digit codes

ISCO08 2-digit	Occupation	Remote work feasibility index	Social interaction index
11	Chief Executives, Senior Officials and Legislators	0,972	0,826
12	Administrative and Commercial Managers	0,923	0,505
13	Production and Specialized Services Managers	0,693	0,513
14	Hospitality, Retail and Other Services Managers	0,698	0,932
21	Science and Engineering Professionals	0,584	0,194
22	Health Professionals	0	1
23	Teaching Professionals	0,427	0,875
24	Business and Administration Professionals	0,901	0,330
25	Information and Communications Technology Professionals	1	0
26	Legal, Social and Cultural Professionals	0,530	0,895
32	Health Associate Professionals	0,067	0,944
33	Business and Administration Associate Professionals	0,783	0,750
34	Legal, Social, Cultural and Related Associate Professionals	0,236	0,963
35	Information and Communications Technicians	0,852	0,570
41	General and Keyboard Clerks	1	0,557
42	Customer Services Clerks	0,478	0,942
43	Numerical and Material Recording Clerks	0,554	0,677
44	Other Clerical Support Workers	0,245	0,850
51	Personal Services Workers	0,012	0,889
52	Sales Workers	0,142	1
53	Personal Care Workers	0,005	1
54	Protective Services Workers	0	1
61	Market-oriented Skilled Agricultural Workers	0	0,452
62	Market-oriented Skilled Forestry, Fishery and HuntingWorkers	0	0,268
63	Subsistence Farmers, Fishers, Hunters and Gatherers	0	0,558
71	Building and Related Trades Workers (excluding Electricians)	0	0,909
72	Metal, Machinery and Related Trades Workers	0	0,304
73	Handicraft and Printing Workers	0,095	0,280
74	Electrical and Electronic Trades Workers	0	0,975
75	Food Processing, Woodworking, Garment and Other Craft and…	0	0,748
81	Stationary Plant and Machine Operators	0	0,272
82	Assemblers	0	0,444
83	Drivers and Mobile Plant Operators	0	0,792
91	Cleaners and Helpers	0	0,389
92	Agricultural, Forestry and Fishery Labourers	0	0,933
93	Labourers in Mining, Construction, Manufacturing and Transport	0	0,823
94	Food Preparation Assistants	0	1
95	Street and Related Sales and Services Workers	1	1
96	Refuse Workers and Other Elementary Workers	0,009	0,957

Data: Preliminary SHARE wave 8 release 0. Conclusions are preliminary. Notes: the two indexes have been generated on ISCO08 2-digit codes. Code 32 (Science and Engineering Associate Professionals) is missing.

Table 4. Tobit regression model – Weeks of interruption

	Baseline model	*Full model*
Essential Jobs	-3.180***	-1.529***
	(0.515)	(0.545)
Remote work feasibility index	-6.038***	-3.886***
	(0.680)	(0.744)
Social interaction index	0.609	1.940**
	(0.721)	(0.753)
Essential_RemoteWorkIndex	Yes	Yes
Essential_SocialInteractionIndex	Yes	Yes
Country dummies	Yes	Yes
Additional covariates	No	Yes
N	7619	6878
Pseudo-r2	0.0437	0.0515
Log pseudolikelihood	-7314.13	-6646.81

Data: Preliminary SHARE wave 8 release 0. Conclusions are preliminary. Notes: in the *baseline* model 6269 observations are left-censored, while 1350 are uncensored. In the *full* model 5636 observations are left-censored, while 1242 are uncensored. (* p<0.1, **p<0.05, ***p<0.01)

Fig. 1 Sample composition by age group and country

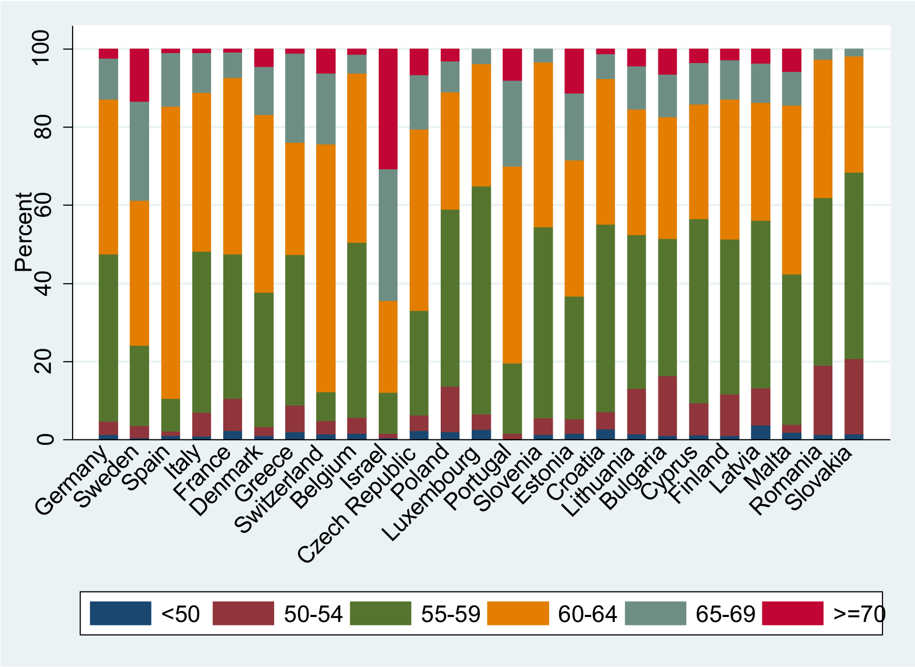

Data: Preliminary SHARE wave 8 release 0. Conclusions are preliminary
